# The workload for home visits by German family practitioners: an analysis of regional variation in a cross-sectional study

**DOI:** 10.1186/s12875-018-0891-6

**Published:** 2019-01-04

**Authors:** Maik Pochert, Karen Voigt, Martin Bortz, Alessa Sattler, Jeannine Schübel, Antje Bergmann

**Affiliations:** Department of General Practice/Medical Clinic III, University Hospital Carl Gustav Carus of the Technische Universität Dresden, Fetscherstraße 74, 01307 Dresden, Germany

**Keywords:** Home visits, Cross-sectional study, Workload, Family practitioners, Rural-urban, Homebound patients

## Abstract

**Background:**

In Germany, home visits account for a considerable workload for many family practitioners, substantial rural-urban disparities are assumable with regards to home visit frequency and duration. Considering the ongoing demographic change and a rural-urban migration a significant regional difference in the provision of care is assumable. There is a lack of reliable data on the current provision of home visits and how their organisational procedures can be ensured in the future. The aim of this study was to describe and assess the average workload of family practitioners during home visits and compare their rural-urban variations.

**Methods:**

A cross-sectional study over a period of 12 months was conducted in Saxony as part of the fifth project of the Saxon Epidemiologic Studies in General Practice (SESAM-5). Over a 1-week period, family practitioners documented every home visit and answered questionnaires about sociodemographic, organisational and clinical characteristics. According to common categorizations in empirical studies four regional groups (rural, semi-rural, semi-urban, urban) were analysed and compared by non-parametric tests: Kruskal-Wallis followed by Dunn’s, Jonckheere-Terpstra and Mann-Whitney-U. Multinomial logistic regression analyses were carried out using a collection of plausible predictors to assess influences for a high frequency and a long duration of home visits.

**Results:**

The sample included 3673 home visits conducted by 253 family practitioners. On average, 14.5 home visits were carried out per week with an average duration of almost 28 min. After comparing regional areas, the number and total duration per home visit showed significant differences between the regions: 8.2 h (rural), 7 h (semi-rural), 6.6 h (semi-urban) and 5 h (urban). The regression analyses found that a high frequency of home visits was most likely accomplished in rural regions and a long duration was most likely performed in private homes.

**Conclusions:**

Workload of home visits is strongly associated with the regional location of the practice, leading to rural-urban disparities. Strategies to reduce regional disparities to ensure the future provision of care in the German and comparable health care systems should be discussed, e.g. by financial incentives (short-term), exploiting the potential of delegation (medium-term) and discussing the implementation of substitution (long-term).

## Background

In Germany, where primary care is not offered by communities, home visits of family practitioners (FP) remain the common method of ensuring low-threshold health care access for immobilized patients [1]. This is particularly relevant for the growing group of elderly living in single households in rural areas [2]. Here, home visits empower a lifestyle that is to a high degree self-determined and prevent or delay the need for hospitalizations or professional long-term care [3].

In the last few decades, declines in home visits by physicians have been reported in most industrialized countries, e.g. Germany, United Kingdom, Netherlands or Australia [4–6]. In this context, it is important to review two developing challenges to ensure the future access of primary care for homebound patients. First, the sociodemographic transition is leading to growing proportions of (frail) elderly, resulting in rising numbers of chronic diseases, multimorbidity and age-related immobility [1, 2]. Mainly, home care for elderly people is provided informally by life partners and adult children, but social changes are weakening this social support network, e.g. caused by increasing professional and private mobility leading to a higher geographical separation between parents and children [7, 8]. Second, the regional maldistribution of FP results in a rising shortage even in the shrinking rural areas [9, 10]. Caused by rising numbers of medical students and graduates choosing specialist medical disciplines [11]. And in addition, the growing urbanization boom is attracting young people with better transport, social and cultural infrastructure to urban areas while decreasing the attractiveness of rural regions [9, 12]. Both developing challenges are particularly important for the workload of home visits, as the decreasing numbers of FP will increase the workload of the remaining FP in rural regions, which will be faced with a rising numbers of time-consuming home visits, as studies show that elderly people utilize more home visits, especially over 75 years [1, 2, 4, 6, 9, 10].

In the German primary care system, home visits are predominantly delivered by FP working solo or in small practices (like in Belgium or the Czech Republic), and in contrast to more team-oriented practices (e.g. United States, United Kingdom or Sweden), there is no broad accessibility of mid-level practitioners qualified to conduct home visits on a regular basis [13, 14]. Based on surveys, German FP spend between 5 to 8 h per week for home visits with big variations in which 10% spend less than 2 h and 15% more than 12 h per week [15, 16]. Those data support that home visits of German FP continue to be a considerable factor in the workload, especially for some FP with higher frequency and duration of home visits [17]. International studies suggest that those differences could be derived from regional variations, but current research in Germany lacks representative data about time requirements and disparities in home visits [6, 18–23]. Those data are needed to assist in the ongoing discussion in Germany, which assumes that the utilization of home visits is disproportionately higher in rural regions [17]. To fill this research gap and assist in the ongoing debate a cross-sectional study was conducted in the German federal state of Saxony. Saxony is a mixed rural and urban area with approx. 4.082 million inhabitants on 18,450 km^2^ ranging from 98 (rural) to 1918 (urban) inhabitants per square kilometres [24]. With 24.9% of the population being 65 years or older the demographic profile is characterized by one of the highest proportions of elderly within Germany as well as Europe [25]. Especially, the Saxonian rural regions show high proportions of average ages, young adult emigration and single or childless households [23]. More than two-thirds of these regions show FP shortages with increasing difficulties to replace an ageing medical workforce, furthermore 28% of FP are 60 years or above and will retire in the coming years [26, 27].

Our primary objectives in this study were to 1) determine the average time requirements of home visits among four regional areas, and 2) assess predictors for a high frequency and a long duration of home visits. The results aim to 1) provide lacking research data, 2) to test the prespecified hypothesis of rural-urban disparities in the field of home visits, and to 3) provide implications for health policy to ensure future primary care access for homebound patients living in ageing societies, especially in rural or underserved regions, as well for other countries where home visits are also a basic task in the daily work life of FP.

## Methods

### Ethics

The study was approved by the ethics committee of the Technical University of Dresden (EK 350092013) and adheres to the Declaration of Helsinki. Written consent was obtained from every participant (FP, assistant physicians, medical assistants) and their data were collected pseudonymously. According to national guidelines a patient consent was not required because patient data were documented anonymously.

### Study design

We conducted a cross-sectional study between July 2014 until June 2015 to investigate the content and organisational characteristics of home visits in family practices in Saxony. The study is named “SESAM-5” because it is the fifth project of the Saxon Epidemiologic Studies in General Practice, initiated and coordinated by the Saxon Association of General Practice in cooperation with the Department of General Practice at the Technical University of Dresden. The design is based on the evaluation of a preceding feasibility study, which analysed the study design including recruitment strategies and study instruments [28]. Prior to the start of the SESAM-5, target group-specific advertisements in journals including an online information source (http://www.sesam-studien.de) were used to increase the recruitment rate.

### Study setting and participants

In March 2014, an invitation to participate was mailed to all 2677 registered FP in Saxony. Contact information was obtained from an official register of the Saxon Association of Statutory Health Insurance Physicians, which is a permanently updated register in which every FP is mandatory to register. With this contact information we were able to invite the entire population of FPs providing outpatient primary care in Saxony, regardless of their involvement in the field of home visits.

From 01 July 2014 to 31 June 2015, every participating family physician was randomly allocated a documentation week by a pseudo random number generator regarding their opening hours. In this 1-week period, every single home visit was documented by the person performing the home visit. Therefore, they fill out on their own for every home visit a standardized documentation with a semi-structured questionnaire that included closed and open questions regarding organisational features and the content of the home visit, as well as sociodemographic and clinical characteristics of the patient. Furthermore, information about sociodemographic and organisational characteristics of the medical practice were requested from every FP and medical assistant that conducted home visits. Answers concerning open-ended variables were classified based on contents targeting the integration of common categories in statistical analysis. A 12-month period was chosen to avoid seasonal biases, and a 1-week period to meet the organisational characteristics of the individual home visit plans.

### Variables

To describe the time requirements of home visits five variables were used: 1) the *frequency per week* counted the number of every home visit of each FP in the documentation week; 2) the *treatment time in minutes (min.)* quantified the time of treatment for each home visits, without including further tasks after the home visits, e.g. documentation of the visits in the medical record; 3) the *travel time in min.* Accounted for the amount of time for the round trip back to the patient regardless of the kind of traveling (driving, public transportation etc.), for multiple visits the travel time was divided by the number of patients, e.g. 30 min. to visit a nursing home with three patients would account for 10 min. Travel time for each patient; 4) the *total duration in min.* Was calculated by the addition of the treatment time plus the travel time; 5) the weekly workload was calculated by the multiplication of the total duration with the frequency per week.

To disaggregate for geographical areas, data were categorized by regional distribution, and allocated into four groups depending on the number of inhabitants of Saxony according to common used differentiations [29]: 1) rural areas are defined by fewer than 5000 inhabitants, reflecting 16% of the population living in 255 rural communities; 2) semi-rural areas are defined by 5000–10,000 inhabitants, reflecting 17% of the population living in 99 small towns; 3) semi-urban areas are defined by 10,000–50,000 inhabitants, reflecting 28.5% of the population living in 63 medium-sized towns; and 4) urban areas are defined by over 50,000 inhabitants, reflecting 38.5% of the population living in the six largest cities [24, 30]. To disaggregate for primary care status, data were categorized into two groups based on the classification of the Association of Statutory Health Insurance Physicians, which calculates the primary care status on a fixed ratio between the numbers of physicians in practice and inhabitants of a defined region [31]: 1) well-served regions are supplied with a ratio over 100%; 2) imminent underserved regions are supplied with a ratio between 75 to 100%.

### Statistical analysis

Descriptive statistics including percentages, mean, standard deviation (± SD), 95% confidence intervals (95% CI), and minimum to maximum (min – max) were used to describe the FP and patient characteristics. Also, the time requirements for home visits were described (frequency, treatment time, travel time, total duration and weekly workload) and compared according to regional area. Criteria for parametric tests were not met, because variables showed significant outliers and failed tests for normality. In addition, three variables (treatment time, travel time and total duration) revealed a lack of homogeneity of variances. Therefore, non-parametric tests were used: Kruskal-Wallis H test was followed by Dunn’s (1964) test procedure with a Bonferroni correction for multiple comparisons. The non-parametric Jonckheere-Terpstra test was used to determine if there is a statistically significant difference between the regions. Comparisons between just two regions was done by non-parametric Mann-Whitney-U test.

Regression analyses were performed to examine potential predictors that may impact the workload for home visits of FP. For the purpose of investigation, the workload for home visits was divided into two parts: 1) the average frequency per week, which was assessed on the practice level and 2) the average duration per home visit (treatment plus travel time), which was assessed on the individual patient level**.** For the regression analyses, we wanted to use linear regression, but it violates the linearity assumptions [32]. Then we tended to use ordinal logistic regression, but it violates the proportional odds assumption, which might result in an incorrect conclusion [33]. Finally, we selected multinomial logistic regression models instead given its less binding assumptions. To perform those models, it was necessary to build categorically distributed dependent variables, therefore we categorize both variables into their terciles: frequency (low, average, high) and duration (short, average, long). To assess the models, we provide overall goodness-of-fit tests and likelihood-ratio test to compare each full model to the intercept-only model. A *p*-value of 0.05 was considered significant. All analyses for this study were conducted using the statistical program IBM SPSS Statistics 23.0.

## Results

### Participants

**Fig. 1:**
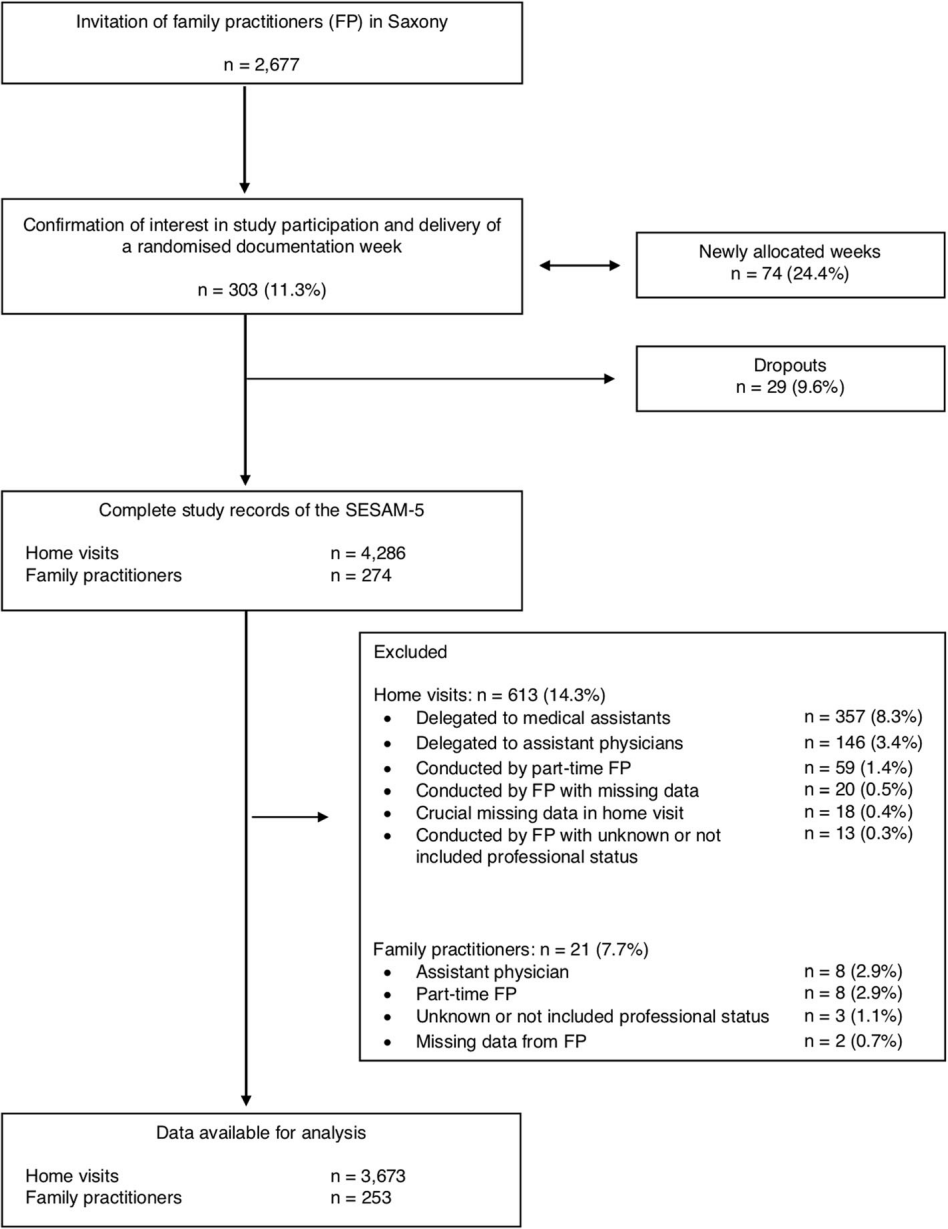
Flow diagram of the SESAM-5

A flow diagram of the SESAM-5 (Fig. 1) summarizes the recruitment process. 303 of 2677 (11.3%) potentially assessed eligible and invited FP expressed their interest in participating. All 303 were examined for eligibility and allocated a randomised documentation week. In 74 cases (24.4%), a new documentation week was necessary. Ultimately, 274 of 303 (90.4%) FP returned the documentation forms and were included in the SESAM-5. The main reason for the 29 (9.6%) dropouts was illness, death, huge workload, or no further interest. A total number of 274 FP were included, and 4286 home visits were documented in the SESAM-5. For the present paper, only home visits carried out by FP working full-time were analysed, because they fulfil the study design criteria for the entire 1-week period. Of those, 613 (14.3%) home visits were excluded from the analysis, since 357 (8.3%) were delegated to medical assistants, 146 (3.4%) were delegated to assistant physicians, 59 (1.4%) were conducted by part-time FP, 20 (0.5%) were conducted by FP with missing data, 13 (0.3%) were conducted by FP with unknown or not included professional status and 18 (0.4%) represented the missing rate. Of the FP, 21 (7.7%) dropped out, since 8 (2.9%) were assistant physicians, 8 (2.9%) worked part-time, 3 (1.1%) had unknown or not included professional status and another 2 (0.7%) had missing data. Table 1 presents the characteristics of the FP involved by region.

**Table 1:** Family practitioner characteristics by region

Characteristics	Regions					
	Saxony	All	Rural < 5000	Semi-rural 5000 – 10,000	Semi-urban 10,000 – 50,000	Urban > 50,000
N	2704	253	68	44	78	63
Gender^c^						
Female	61.4%^d^	62.1%	61.8%_a_	54.5%_a_	59%_a_	71.4%_a_
Male	38.6%	36.4%	36.8%_a_	38.6%_a_	41%_a_	28.6%_a_
Age						
Mean (± SD)	53	51.6 (± 8.6)	53.2* (± 8.3)	50.8 (± 8)	52.2 (± 8.7)	49.8 (± 9.1)
39 years and younger	7.9%	10.3%	5.9%	6.8%	12.8%	14.3%
40–49	27.2%	27.3%	25%	40.9%	14.1%	36.5%
50–59	37.8%	41.9%	47.1%	34.1%	51.3%	30.2%
60 years and older	27.2%	18.2%	22.1%	15.9%	16.7%	17.5%
Practice type^c^						
Single-FP practice	67.7%	64.4%	76.5%_a_	63.6%_a, b_	65.4%_a, b_	50.8%_b_
Multi-FP practice^f^	32.3%	35.2%	22%_a_	36.3%_a, b_	34.6%_a, b_	49,2%_b_
Primary care status^f^						
Well-served regions	64.9%	69.6%	63.2%_a, b_	54.5%_b_	74.4%_a, b_	81%_a_
Imminent underserved	35.1%	30.4%	36.8%_a, b_	45.5%_b_	25.6%_a, b_	19%_a_
Share of delegation^e^	unknown	12.1%	7%*	11.9%	15.3%	14.7%

*Note:* Pairwise regional comparisons with the urban region (> 50,000) were depicted with: *** *p* < 0.001; ** *p* < 0.01; * *p* < 0.05

^c^Pairwise regional comparisons, in which each subscript letter denotes a subset of intervention categories whose column proportions do not differ significantly from each other at the 0.05 level

^d^Working with two or more FP in one practice

^e^Other pairwise comparisons revealed statistically significant differences in the share of delegation (to medical or non-medical colleagues) between the rural (< 5000) and semi-urban region (10,000 – 50,000) (*p* < 0.05)

^f^Not all totals add up to 100% because not all respondents answered every question

The sample was representative of the FP population of Saxony for characteristics such as gender, middle age (40–49 and 50–59) in the assignment to underserved regions and practice share. But the sample was younger in age especially the age group with 60 years and older, which could limit the external validity (cf. Strengths and limitations). The share of delegation in Saxony was unknown, but in the study the share was lower by FP in (semi)rural backgrounds compared to (semi)urban backgrounds.

### Characteristics of homebound patients by region

Table 2 presents the homebound patient characteristics by region. Around two-thirds of all home visits were carried out for female and one-third for male patients. The mean age was 82.3 (± 11.2) years. The average number of chronic diseases was 6.4 (± 3.9) per patient and decreased from rural to urban setting. On average, 56% of the homebound patients lived in private homes (alone in one’s own apartment or living with family or partner) and 41.4% lived in long-term care institutions. The share of home visits conducted in private homes decreased with increasing regional population; in contrast, in long-term care institutions the share increased with rising regional populations.

**Table 2:** Characteristics of homebound patients by region

Characteristics	Regions				
	All	Rural < 5000	Semi-rural 5000 – 10,000	Semi-urban 10,000 – 50,000	Urban > 50,000
N	3673	1119	643	1123	788
Gender^e^					
Female	66.8%^f^	65.7%_a_	68%_a_	66.8%_a_	67.4%_a_
Male	31.9%	33.6%_a_	31.6%_a_	32.2%_a_	29.4%_a_
Age^g^					
Mean (± SD)	82.3 (± 11.2)	82.6 (± 11.7)	82.2 (± 10.8)	81.6 (± 11.6)	82.7 (± 10.3)
95% CI	81.9–82.6	81.9–83.3	81.4–83.1	80.9–82.3	82–83.5
Min – Max	2–104	2–104	5–102	27–102	24–101
Chronic diseases^h^					
Mean (± SD)	6.4 (± 3.9)	7*** (± 4)	6.7*** (± 4.1)	6** (± 3.7)	5.8 (± 4)
95% CI	6.3–6.5	6.8–7.2	6.4–7	5.8–6.2	5.6–6.1
Min – Max	1–36	1–29	1–31	1–35	1–36
Housing situation^e^					
Private homes^i^	56%	66.4%_a_	61.6%_b_	52.8%_c_	41.4%_d_
Long-term care institutions	41.4%	30.7%_a_	37.5%_b_	45.2%_c_	54.4%_d_
Type of home visit^e^					
Planned	69.1%	68.6%_a_	66.4%_a_	66.3%_a_	75.9%_b_
Requested	30%	30.7%	33.4%_a_	33.2%_a_	21.6%_b_

Note: Pairwise regional comparisons with the urban region (> 50,000) were depicted with: *** *p* < 0.001; ** *p* < 0.01; * *p* < 0.05

^e^Pairwise regional comparisons, in which each subscript letter denotes a subset of intervention categories whose column proportions do not differ significantly from each other at the 0.05 level

^f^Not all totals add up to 100% because not all respondents answered every question

^g^Other pairwise comparisons revealed statistically significant differences between: rural and semi-urban region (*p* < 0.05)

^h^Other pairwise comparisons revealed statistically significant differences between: rural and semi-rural region (*p* < 0.05); rural and semi-urban region (*p* < 0.001); semi-rural regions and semi-urban region (*p* < 0.001)

^i^Living (alone or with family or partner) in own apartment

### Comparison of time requirements of home visits by regional distribution

Table 3 compares the time requirements of home visits by regional distribution. Overall, the average *frequency of home visits per week* was 14.5, with an average time per home visit of 27.8 min divided into 14.8 min of treatment and 13 min of travel time, resulting in a calculated weekly workload of 6.7 h. The weekly frequency of home visits decreased statistically significant with rising regional populations (*p* <  0.001) and was significantly higher in rural with 16.5 than in urban regions with 12.5 (*p* < 0.001).

**Table 3:** Comparison of time requirements of home visits by regional distribution

Home vists	Regions					
	All	Rural < 5000	Semi-rural 5000 – 10,000	Semi-urban 10,000 – 50,000	Urban > 50,000	*P*-value _TREND_^a^
N	3673	1119	643	1123	788	
Frequency per week						< 0.001
Mean (± SD)	14.5 (± 11.3)	16.5*** (± 10.4)	14.6* (± 8.6)	14.4* (± 9.7)	12.5 (± 15.1)	
95% CI	13.1–15.9	13.9–19	12–17.2	12.2–16.6	8.7–16.3	
Min – Max	0–97	1–55	2–51	0–62	0–97	
Treatment time in min.^b^						< 0.001
Mean (± SD)	14.8 (± 9.3)	15*** (± 8.8)	16.2*** (± 9.9)	15.1*** (± 9.9)	12.8 (± 7.6)	
95% CI	14.5–15.1	14.5–15.5	15.4–17	14.5–15.6	12.2–13.3	
Min – Max	1–110	2–60	2–88	2–78	1–110	
Travel time in min.						< 0.001
Mean (± SD)	13 (± 14.3)	14.9*** (± 18.2)	12.4** (± 11.2)	12.6* (± 12.8)	11.4 (± 11.7)	
95% CI	12.5–13.5	13.8–15.9	11.6–13.3	11.9–13.4	10.6–12.2	
Min – Max	0–200	0–200	0–80	0–170	0–70	
Total duration in min.						< 0.001
Mean (± SD)	27.8 (± 18.9)	29.9*** (± 21.8)	28.6*** (± 16.6)	27.7*** (± 18.1)	24.2 (± 16.5)	
95% CI	27.2–28.4	28.6–31.2	27.4–30	26.6–28.8	23–25.3	
Min – Max	2–240	2–240	2–110	2–232	3–126	
Weekly workload	6.7 h	8.2*** h	7* h	6.6 h	5 h	< 0.001
Ratio	1 (reference)	1.22	1.04	0.99	0.75	
Share of treatment time	53.2%	50.2%	56.6%	54.5%	52.9%	
Share of travel time	46.8%	49.8%	43.4%	45.5%	47.1%	

Note: Pairwise regional comparisons with the urban region (> 50,000) were depicted with: *** *p* < 0.001; ** *p* < 0.01; * *p* < 0.05

^a^Comparisons between all regions with the Jonckheere-Terpstra test

^b^Other pairwise comparisons revealed statistically significant differences in treatment time between the rural and semi-rural region

(*p* < 0.05), and between the semi-rural region and semi-urban region (*p* < 0.05)

The *treatment time per home visit* was highest in semi-rural areas with 16.2 min and was significantly lower in urban regions with 12.8 min (*p* < 0.001). The *travel time per home visit* was significantly lower in urban areas at 11.4 min, exactly 3.5 min less compared to the rural regions with 14.9 min (*p* < 0.001). The averages of the *total duration per home visit* showed a significantly decreasing correlation with an increasing number of inhabitants (*p* <  0.001): rural regions peaked at 29.9 min, followed by semi-rural areas at 28.6 min; semi-urban areas amounted at 27.7 min and lastly urban regions at 24.2 min per home visit. Pairwise comparisons revealed statistically significant differences in the total duration between the urban region: 1) with the rural region (*p* < 0.001); 2) with the semi-rural region (*p* < 0.001); and 3) with the semi-urban region (*p* < 0.001), but not between any other group combination.

In addition, the calculated *weekly workload for home visits* confirmed a significant correlation with a decreasing workload with an increasing number of inhabitants (*p* < 0.001): in rural regions, the weekly workload amounted to over 8 h, and was half distributed to travel time (49.8%); in semi-rural and semi-urban regions, it ranged between 7 to 6.6 h, and was more distributed by treatment time (56.6% vs. 54.5%), and lastly, in urban regions it was the lowest, at 5 h with a tight majority share of treatment time (52.9%). In direct comparison, the weekly workload in rural regions was with more than 3 h significantly higher than in urban regions, resulting in an almost 40% lower workload for urban colleagues in the field of home visits.

### Multinomial logistic regression analysis of influencing factors on the ***frequency of home visits***

Table 4 shows the multinomial logistic regression analysis, which was run to determine the effects of:

**Table 4:** Analysis of influencing factors on the frequency of home visits

Frequency of home visits per week	B	SE	Wald	df	p	Odds Ratio	95% CI for Odds Ratio	
							Lower	Upper
AVERAGE^a^ (10 to 16 home visits)								
1) Regional distribution^b^								
Rural (< 5000)	1.630	.521	9.772	1	.002**	5.104	1.837	14.183
Semi-rural (5000 – 10,000)	1.963	.568	11.950	1	.000***	7.118	2.339	21.659
Semi-urban (10,000 – 50,000)	1.913	.493	15.045	1	.000***	6.772	2.576	17.802
2) Regional primary care status								
Imminent underserved regions^c^	−.088	.408	.047	1	.829	.915	.412	2.036
3) Overall patient population								
Patient contacts per quarter	.000	.001	.050	1	.824	1.000	.999	1.001
Share of patients over 65 years	−.009	.012	.616	1	.433	.991	.969	1.014
4) Organizational characteristics								
Share of delegation	−.022	.009	5.696	1	.017*	.978	.960	.996
HIGH^a^ (17 or more home visits)								
1) Regional distribution^b^								
Rural (< 5000)	1.249	.479	6.807	1	.009**	3.488	1.365	8.918
Semi-rural (5000 – 10,000)	1.031	.543	3.611	1	.057	2.803	.968	8.119
Semi-urban (10,000 – 50,000)	1.007	.461	4.778	1	.029*	2.738	1.110	6.755
2) Regional primary care status								
Imminent underserved regions^c^	.450	.386	1.359	1	.244	1.568	.736	3.338
3) Overall patient population								
Patient contacts per quarter	.001	.000	7.021	1	.008**	1.001	1.000	1.002
Share of patients over 65 years	.005	.011	.208	1	.648	1.005	.984	1.027
4) Organizational characteristics								
Share of delegation	−.037	.012	10.060	1	.002**	.964	.943	.986

*Note:* *** *p* < 0.001; ** *p* < 0.01; * *p* < 0.05

^a^The reference category is: low with 9 home visits or less

^b^Each region (rural, semi-rural and semi-urban) is compared to urban regions

^c^Imminent underserved regions are compared to well-served regions

1) regional distribution (rural, semi-rural, semi-urban, urban),

2) regional primary care status (well-served regions, imminent underserved regions),

3) overall patient population (patient contacts per quarter, share of patients over 65 years) and

4) organizational characteristics (share of delegation),

on the likelihood of a low, average or high weekly frequency of home visits. The goodness-of-fit tests indicated that the model was a good fit to the observed data (Pearson: χ^2^(430) = 446.920, *p* = 0.277; deviance: χ^2^(430) = 440.638, *p* = 0.351), but most cells were sparse with zero frequencies in 65.6% of cells. However, the final model statistically significantly predicted the dependent variable over and above the intercept-only model (χ^2^(14) = 55.144, Nagelkerke *R*^2^ = 0.235, *p* <  0.001). The model contained three groups (tercile), in which an average frequency (10 to 16 home visits) and a high frequency (17 or more home visits) was each compared to the reference category of a low frequency (9 or less home visit) – the following predictor variables were statistically significant:

1. Related to urban regions, the odds of conducting an average instead of a low frequency increased:

- 5.104 Times (OR) in rural regions (*p* < 0.01),
- 7.118 Times (OR) in semi-rural regions (*p* < 0.001),
- 6.772 Times (OR) in semi-urban regions (*p* < 0.001),

and furthermore, the odds of conducting a high instead of a low frequency increased:

- 3.488 (OR) times higher in rural regions (*p* < 0.01) and
- 2.738 (OR) times higher in semi-urban regions (*p* < 0.05).

2) The predictor variables of the regional primary care status were not statistically significant.
3) Each additional patient contact increased the odds of a high frequency of home visits by 0.1% (OR = 1.001; *p* < 0.01).
4) A 1 % rise in the share of delegation to medical or non-medical colleagues decreased the odds of conducting an average frequency of home visit by 2.2% (OR = 0.978; *p* < 0.05) and the odds of a high frequency of home visits by 3.6% (OR = 0.964; *p* < 0.01).

### Multinomial logistic regression analysis of influencing factors on the ***duration of home visits***

Table 5 shows the multinomial logistic regression analysis, which was run to determine the effects of:

1. regional distribution (rural, semi-rural, semi-urban, urban),
2. regional primary care status (well-served regions, imminent underserved regions),
3. homebound patient characteristics (gender, age, number of chronic diseases) and
4. home visit characteristics (housing situation, type of home visit),

**Table 5:** Analysis of influencing factors on the duration of home visits

Duration of home visits	B	SE	Wald	df	p	Odds Ratio	95% CI for Odds Ratio	
							Lower	Upper
AVERAGE^a^ (20 to 30 min)								
1) Regional distribution^b^								
Rural (< 5000)	.160	.137	1.360	1	.244	1.173	.897	1.534
Semi-rural (5000 – 10,000)	.359	.154	5.414	1	.020*	1.431	1.058	1.936
Semi-urban (10,000 – 50,000)	.253	.132	3.672	1	.055	1.288	.994	1.669
2) Regional primary care status								
Imminent underserved regions^c^	.285	.102	7.891	1	.005**	1.330	1.090	1.623
3) Homebound patient characteristics								
Gender^d^	−.070	.105	.444	1	.505	.933	.759	1.145
Age	.014	.005	9.074	1	.003**	1.014	1.005	1.023
Number of chronic diseases	.019	.012	2.517	1	.113	1.019	.996	1.043
4) Home visit characteristics								
Housing situation^e^	1.410	.098	206.600	1	.000***	4.094	3.378	4.962
Type of home visit^f^	.718	.113	40.537	1	.000***	2.051	1.644	2.559
LONG^a^ (31 min or longer)								
1) Regional distribution^b^								
Rural (< 5000)	.022	.126	.030	1	.862	1.022	.799	1.307
Semi-rural (5000 – 10,000)	.181	.144	1.566	1	.211	1.198	.903	1.589
Semi-urban (10,000 – 50,000)	.056	.122	.213	1	.644	1.058	.833	1.344
2) Regional primary care status								
Imminent underserved regions^c^	.107	.096	1.229	1	.268	1.113	.921	1.344
3) Homebound patient characteristics								
Gender^d^	.040	.097	.169	1	.681	1.041	.861	1.258
Age	.011	.004	6.549	1	.010*	1.011	1.003	1.019
Number of chronic diseases	.014	.011	1.418	1	.234	1.014	.991	1.036
4) Home visit characteristics								
Housing situation^e^	1.863	.093	403.501	1	.000***	6.443	5.372	7.728
Type of home visit^f^	1.256	.103	147.627	1	.000***	3.511	2.867	4.300

*Note:* *** *p* < 0.001; ** *p* < 0.01; * *p* < 0.05

^a^The reference category is: short with 19 min or less

^b^Each region (rural, semi-rural and semi-urban) is compared to urban regions

^c^Imminent underserved regions are compared to well-served regions

^d^Gender is for males compared to women

^e^Housing situation is for private homes compared to long-term care institutions

^f^Type of home visit is for requested home visits compared to planned home visits

on the likelihood of a short, average or long duration of home visits. The goodness-of-fit tests indicated that the model was a good fit to the observed data (Pearson: χ^2^(5894) = 6006.748, *p* = 0.150; deviance: χ^2^(430) = 6003.295, *p* = 0.157), but most cells were sparse with zero frequencies in 63.8% of cells. However, the final model statistically significantly predicted the dependent variable over and above the intercept-only model (χ^2^(18) = 692.066, Nagelkerke *R*^2^ = 0.205, *p* < 0.001). The model contained three groups (tercile), in which an average duration (20 to 30 min) and a long duration (31 min or longer) was each compared to the reference category of a short duration (19 min or less) – the following predictor variables were statistically significant:

1. Related to urban regions, the odds of conducting an average instead of a short duration increased 1.431 times (OR) in semi-rural regions (*p* < 0.01).
2. Compared to well-served regions, the odds of conducting an average instead of a short duration increased by 33% in imminent underserved regions (OR = 1.330; *p* < 0.01).
3. Older patient populations increased the odds of an average duration of home visits by 1.4% (OR = 1.014; *p* < 0.01) and the odds of a long duration by 1.1% (OR = 1.011; *p* < 0.01).
4. Related to long-term care institutions, the odds of conducting an average instead of a short duration increased 4.094 times (OR) in private homes (*p* < 0.001), and furthermore, the odds of a high instead of a short duration increased 6.443 times (OR) in private homes (*p* < 0.001). Compared to planned home visits, the odds of conducting an average instead of a short duration on requested home visits increased 2.051 times (OR) (*p* < 0.001), and furthermore the odds of a high instead of a short duration on requested home visits increased 3.511 times (OR) (*p* < 0.001).

## Discussion

This study evaluated the time requirements of home visits that were found to be strongly associated with regional distribution. The average frequency, the total duration and weekly workload of home visits decreased with an increasing number of inhabitants and was significantly higher in rural than in urban regions. The multinomial logistic regression analyses found different predictors influencing the frequencies and durations of home visits.

### Frequency of home visits per week

#### Patient contacts and share of delegation

FP with more patient contacts and the tendency to use the delegation less were more likely to conduct a higher frequency of home visits. Those results can be explained by the fact that a total of more treated patients increases the probability to conduct more home visits, and a higher use of delegation reduces the likelihood of more visits.

#### Regional distribution

FP from rural compared to urban regions were much more likely to conduct a higher frequency of home visits. The rural-urban disparities may be explained by four aspects. Firstly, the higher proportion of elderly patients with more chronic diseases in rural areas may be correlated to the higher home visit frequency (Table 2). Secondly, a variety of structural changes increases the probabilities of more home visits, e.g. medical facilities are less frequently settled in rural regions leading to lengthy travel distances, and at the same time, rural public transportation systems are diminishing leading to limited travel options. Those development can be challenging particularly for (older) patients with limited ability to drive and with less accessibility of other drivers (e.g. adult children) caused by increasing professional and private mobility [7, 8]. Thirdly, another predictor could be derived from cultural rural-urban disparities in which the close cohesion of the FP in the communities and the tradition of rural home visits lead to a higher tendency to request a FP first compared to urban inhabitants, who may tend to call emergency services or go to the close-by emergency department more frequently [1, 34]. Fourthly, linked to the share of delegation, the rural FP used significantly less the delegation to reduce the frequency of home visits compared to urban colleagues (7% vs. 14,7%; *p* < 0.05; Table 1). Possibly, this behaviour could be partially related to the higher proportion of chronic diseases of the rural homebound patients, which might lead to less uncomplicated cases qualified to delegate. And probably, due to the close cohesion in rural regions, the patients and FP might put more emphasis on personal and social contact, so that a delegation of a home visit is less obvious [1, 34].

### Duration of home visits

#### Older homebound patient population and requested home visits

FP with older patients and more requested home visits were more likely to conduct a long duration of home visits. Those results can be explained by the fact that older patient populations are more likely to have factors that cause an encounter to take more time to complete, like reduced mobility or hard-of-hearing. Also, requested visits have a higher probability to contain cases with higher urgency and unplannable situations compared to planned home visits. An analysis of the type of home visit showed that the requested average home visit, lasts more than 8 min longer, compared to a planned home visit (33.7 min. vs. 25.4 min.). Thereby, was the proportion of requested home visits with 30.7% in rural regions significantly higher compared to 21.6% in urban regions (Table 2).

#### Housing situation

A long duration of home visits was most likely performed in private homes. An analysis of the housing situation showed that the average home visit in a private home, lasts more than 10 min longer, compared to a home visit in a long-term care institution (33.1 min. vs. 21.1 min.). This difference could be attributed in half to the treatment time and in half to the travel time. The shorter treatment duration may be explainable by a better nursing care in the professional setting of long-term care institutions. The lower travel time is explainable by the possibilities of conducting multiple visits in long-term care institutions, which reduce the average travel time of each visit, compared to the time-consuming single visits in private homes. Thereby, was the proportion of longer private home visits with 66.4% in rural regions significantly higher compared to 41.4% in urban regions (Table 2).

### Strengths and limitations

The study was conducted with around 9.5% of Saxony’s FP and a large statistical population with 3673 home visits. Gender, middle-age, practice type and primary care status were representative of the FP population of Saxony, but the external validity could be limited by the younger age, especially due to the significantly lower age group of 60 years and older (27.2% vs. 18.2%). Studies suggest a higher involvement in home visits with this FP age cohort [20, 22]. This could lead to an underestimation of the utilization of home visits. A self-selection bias may influence the study, because every FP that expressed their interest in participating was included, which may be correlated with characteristics that influence the study. For example, FP with higher workloads for home visits might be more motivated to participate, and vice versa, FP with low workloads for home visits might be less motivated to participate. This imaginable self-selection would lead to an overestimation of the time requirements of home visits. The study relied on the quality and completeness of data recorded by FP. In this context, a limitation could be found in the comments section of our questionnaire regarding collecting information about chronic conditions. Here, comparisons with the results of other studies based on the secondary analysis of billing data may be limited because the participating FP of the SESAM-5 did only record diseases as chronic diagnoses that are relevant for their diagnostic or therapeutic procedures. However, we accept this because we wanted to focus on relevant and treated diagnoses rather than just documented ones. Also, billing data could be influenced by FP’s financial interests, resulting in the overestimation or underestimation of several diagnoses. The overall patient population of the FP was done by two estimations of the FP: contacts per quarter and the share of patients over 65 years. Both estimations could be biased by an over- or underestimation. Furthermore, the 1-week of documentation was only a short period of time to reflect the whole organisational characteristics of the individual home visit plans.

## Conclusion

The results of this cross-sectional study showed an average frequency of 14.5 home visits per week with a duration of almost 28 min resulting into a workload of 6.7 h. The workload was approximately spent half on treatment and the other half on travel time. The regional analysis confirmed the prespecified hypothesis of rural-urban disparities and resulted into an almost 40% lower workload devoted for home visits in urban compared to rural regions (5 h vs. 8.2 h) caused by a higher frequency (16.5 vs. 12.5) and a longer duration (29.9 min. vs. 24.2 min). Those findings support other international studies suggesting an association between the region of residence and demand for home visits caused by different patient populations [6, 18–22, 34]. In this context, rural population factors result into higher frequency and durations of home visits, as they include higher proportions of elderly patients needing more and longer visits, lower usage of delegation, longer travel distances, more time-consuming requested visits and single-visits in private homes [6, 18–22, 34]. Those findings are particularly important regarding the challenges from sociodemographic transitions and regional maldistribution predicting an increasing demand for time-consuming home visits with decreasing numbers of FP [1–3, 9, 10]. Those developments are predicted to result into rising overall workloads among the remaining FP in rural regions being already higher today as German data suggest a rural-urban disparity between 2 to 6 h per week [35, 36].

There are several strategies that politicians and public health decision makers should consider in order to support FP with high utilization of time-consuming home visits. In the German primary care system, this can be done by using specific strategies to increase the attractiveness of present and future primary care conditions. In the short-term, a financial incentive in more rural regions can at least provide more attractive compensation for the low reimbursement of home visits (22.59 euros) [37]. In the medium-term, the potential of the delegation of home visits to medical professional groups should be exploited to its full potential [38]. A more team-oriented practices with a broad accessibility of mid-level practitioners qualified to conduct home visits on a regular basis, e.g. in the United States, United Kingdom or Sweden, might be a best practice example for Germany [13, 14]. Also, in the context of growing shortage of FP the focus must emphasize on the appropriate use of clinician time in patient care. In this regard, home visits are highly inefficient as our study showed that FP spend on average more than 3 h per week only on home visit related travel time. Here, the establishment of weekly driver services that bring homebound patients to the family practice could be one measure to reduce high travel times. In the long-term, the substitution of home visits in underserved regions should be discussed intensely in combination with additional possibilities and limitations in the field of telemedicine [39–41]. Our results bring new evidence of rural-urban disparities in the current provision of home visits that will be of high interest for other countries with comparable primary care organisation [13, 14].

Finally, further studies are needed to understand the relation of home visits with financial incentives [42]. Furthermore, more studies are needed to investigate the potentials, requirements and risks in the care of homebound patients (especially in rural or underserved regions) due to the greater use of telemedicine [43, 44], as well as a higher use of interprofessional collaboration in the form of delegation or substitution of tasks [14, 38].

## Abbreviations

FP: Family practitioners
min.: minutes
OR: Odds ratio
SESAM-5: Fifth project of the Saxon Epidemiologic Studies in General Practice

## References

[1] Theile G, Kruschinski C, Buck M, Müller CA, Hummers-Pradier E (2011). Home visits - central to primary care, tradition or an obligation? A qualitative study. BMC Fam Pract.

[2] Christensen K, Doblhammer G, Rau R, Vaupel JW (2009). Ageing populations: the challenges ahead. Lancet.

[3] Gonçalves J, Weaver F. Effects of formal home care on hospitalizations and doctor visits. Int J Health Econ Manag. 2016:1–31. 10.1007/s10754-016-9200-x.

[4] Snijder EA, Kersting M, Theile G, Kruschinski C, Koschak J, Hummers-Pradier E, Junius-Walker U (2007). Home visits in German general practice: findings from routinely collected computer data of 158,000 patients. Gesundheitswesen.

[5] National Health Service (2009). Trends in Consultation Rates in General Practice 1995/1996 to 2008/2009: Analysis of the QResearch Database.

[6] Joyce C, Piterman L (2008). Trends in GP home visits. Aust Fam Physician.

[7] Tesch-Römer C, Wurm S (2006). Lebenssituationen älter werdender und alter Menschen in Deutschland. Bundesgesundheitsbl Gesundheitsforsch Gesundheitsschutz.

[8] Ganong LH, Coleman M, Rothrauff T (2009). Patterns of assistance between adult children and their older parents: resources, responsibilities, and remarriage. J Soc Pers Relatsh.

[9] Weinhold I, Gurtner S (2014). Understanding shortages of sufficient health care in rural areas. Health Policy.

[10] Kringos D, Boerma W, Bourgueil Y, Cartier T, Dedeu T, Hasvold T (2013). The strength of primary care in Europe: an international comparative study. Br J Gen Pract.

[11] Kiolbassa K, Miksch A, Hermann K, Loh A, Szecsenyi J, Joos S, Goetz K (2011). Becoming a general practitioner - what factors have most impact on career choices of medical students?. BMC Fam Pract.

[12] Gurtner S, Werner K (2012). Student grants in Saxony--a successful story?. Gesundheitswesen Bundesverb Arzte Offentlichen Gesundheitsdienstes Ger.

[13] Bourgueil Y, Marek A, Mousquès J (2007). Medical group practice in primary care in six European countries, and the Canadian provinces of Ontario and Quebec: what are the lessons for France. QES.

[14] Frossard LA, Liebich G, Hooker RS, Brooks PM, Robinson L. Introducing physician assistants into new roles: international experiences. Med J Aust. 2008:199–201. 10.5694/j.1326-5377.2008.tb01583.x 18279123

[15] GKV-Spitzenverband: „Praxisöffnungszeiten“ Befragung in Arztpraxen (2009). https://www.gkv-spitzenverband.de/media/dokumente/presse/pressemitteilungen/2011/Forsa-Umfrage_GKV_Praxisoeffnungszeiten_16104.pdf. Accessed 01 Nov 2018.

[16] Bundesvereinigung K (2018). Tabellenband Ärztemonitor 2018 Ergebnisse nach Facharztgruppen.

[17] Heymann R, Weitmann K, Weiß S, Thierfelder D, Fleßa S, Hoffmann W (2009). Bevölkerungsdichte, Altersverteilung und Urbanität als Einflussfaktoren der Hausbesuchshäufigkeit – eine Analyse für Mecklenburg-Vorpommern. Gesundheitswesen..

[18] Aylin P, Majeed A, Cook DG (1996). Home visiting by general practitioners in England and Wales. BMJ.

[19] Sullivan CO, Omar RZ, Forrest CB, Majeed A (2004). Adjusting for case mix and social class in examining variation in home visits between practices. Fam Pract.

[20] Svab I, Kravos A, Vidmar G (2003). Factors influencing home visits in Slovenian general practice. Fam Pract.

[21] Adelman AM, Fredman L, Knight AL (1994). House call practices: a comparison by specialty. J Fam Pract.

[22] Unwin BK, Jerant AF. The home visit. Am Fam Physician. 1999:1481–8. 10524492

[23] Statistisches Landesamt des Freistaates Sachsen. Das Wanderungsverhalten der Bevölkerung in den sächsischen Gemeinden als Spiegel wirtschaftlicher Rahmenbedingungen und territorialer Besonderheiten. 2008. https://www.statistik.sachsen.de/download/300_Voe-Zeitschrift/2008_03_29-42_Kirschke.pdf. Accessed 31 Mar 2018.

[24] Statistisches Landesamt des Freistaates Sachsen. Bevölkerung im Freistaat Sachsen Bevölkerungsbestand und –entwicklung. 2018. https://www.statistik.sachsen.de/download/100_Berichte-A/A_I_3_j15_SN.pdf. Accessed 31 Mar 2018.

[25] Statistisches Bundesamt. Ältere Menschen in Deutschland und der EU. 2011. https://www.destatis.de/DE/Publikationen/Thematisch/Bevoelkerung/Bevoelkerungsstand/BlickpunktAeltereMenschen1021221119004.pdf?__blob=publicationFile. Accessed 31 Mar 2018.

[26] Wissenschaftliches Institut der AOK. Ärzteatlas 2016 Daten zur Versorgungsdichte von VertragsÄrzten. 2016. https://www.wido.de/fileadmin/wido/downloads/pdf_ambulaten_versorg/wido_amb_pub-aerzteatlas2016_0716.pdf. Accessed 31 Mar 2018.

[27] Kopetsch T. Dem deutschen Gesundheitswesen gehen die Ärzte aus! Studie zur Altersstruktur und Arztzahlentwicklung. 5. Berlin: Kassenärztliche Bundesvereinigung; 2010.

[28] Voigt K, Bojanowski S, Taché S, Voigt R, Bergmann A (2016). Home visits in primary care: contents and organisation in daily practice. Study protocol of a cross-sectional study. BMJ Open.

[29] Bundesinstitut für Bau-, Stadt-, und Raumforschung. Raumgliederungen auf Gemeindebasis: Stadt- und Gemeindetyp. (2016. http://www.bbsr.bund.de/BBSR/DE/Raumbeobachtung/Raumabgrenzungen/StadtGemeindetyp/download-ref-sgtyp.xlsx?__blob=publicationFile&v=10. Accessed 31 Mar 2018.

[30] Statistisches Landesamt des Freistaates Sachsen. Statistischer Bericht Bevölkerungsentwicklung im Freistaat Sachsen nach Gemeinden I. Halbjahr 2016 A I 2 – hj 1/16. 2017. https://www.statistik.sachsen.de/download/100_Berichte-A/A_I_2_hj1_16_SN.pdf. Accessed 31 Mar 2018.

[31] Gemeinsamer Bundesausschuss (G-BA). Richtlinie des Gemeinsamen Bundesausschusses über die Bedarfsplanung sowie die Maßstäbe zur Feststellung von Überversorgung und Unterversorgung in der vertragsärztlichen Versorgung (Bedarfsplanungs-Richtlinie). 2018. https://www.g-ba.de/downloads/62-492-1624/BPL-RL_2018-02-15_iK-2018-05-12.pdf. Accessed 01 Nov 2018.

[32] Kutner MH, Nachtsheim CJ, Neter J, Li W (2005). Applied linear statistical models.

[33] McCullagh P (1980). Regression models for ordinal data (with discussion). J R Stat Soc.

[34] Boerma WGW, Groenewegen PP (2001). GP home visiting in 18 European countries. Adding the role of health system features. Eur J Gen Pract.

[35] Salzmann A, Hofman W, Heinemann S, Greß S: Wie belastet sind HausÄrztinnen und HausÄrzte in Deutschland? Ein Workload-Vergleich nach Praxislage. 2015. https://fuldok.hs-fulda.de/opus4/frontdoor/deliver/index/docId/353/file/pgp_2015_03_Salzmann+et+al.pdf. Accessed 01 Nov 2018.

[36] Zentralinstitut für die kassenÄrztliche Versorgung. HausÄrzte auf dem Land: Höherer Verdienst bei mehr Stunden und höherem Stundensatz. 2018. https://www.zi.de/fileadmin/images/content/PMs/Zi-PM_Finanzen_Stadt_Umland_Land_2018-10-31.pdf. Accessed 01 Nov 2018.

[37] Kassenärztliche Bundesvereinigung. Einheitlicher Bewertungsmaßstab (EBM) Stand: 1. Quartal. 2018; http://www.kbv.de/media/sp/EBM_Gesamt___Stand_1._Quartal_2018.pdf. Accessed 31 Mar 2018.

[38] Laurant MGH, Hermens RPMG, Braspenning JCC, Sibbald B, Grol RPTM (2004). Impact of nurse practitioners on workload of general practitioners: randomised controlled trial. BMJ.

[39] Laurant M, Reeves D, Hermens R, Braspenning J, Grol R, Sibbald B. Substitution of doctors by nurses in primary care. Cochrane Database Syst Rev. 2005. 10.1002/14651858.CD001271.pub2. 10.1002/14651858.CD001271.pub2 15846614

[40] Dimmick SL, Mustaleski C, Burgiss SG, Welsh T (2000). A case study of benefits & potential savings in rural home telemedicine. Home Healthcare Now.

[41] Jenkins RL, White P. Telehealth advancing nursing practice. Nurs Outlook. 2001. 10.1067/mno.2001.111933. 10.1067/mno.2001.111933 11309565

[42] Flodgren G, Eccles MP, Shepperd S, Scott A, Parmelli E, Beyer FR (2011). An overview of reviews evaluating the effectiveness of financial incentives in changing healthcare professional behaviours and patient outcomes. Cochrane Database Syst Rev.

[43] Koch S (2006). Home telehealth - current state and future trends. Int J Med Inform.

[44] Ekeland AG, Bowes A, Flottorp S (2010). Effectiveness of telemedicine: a systematic review of reviews. Int J Med Inform.

